# Words matter: interpretations and implications of “para” in paraprofessional

**DOI:** 10.5195/jmla.2021.933

**Published:** 2021-01-01

**Authors:** Hannah Schilperoort, Alvaro Quezada, Frances Lezcano

**Affiliations:** 1 schilper@usc.edu, Information Services Librarian, Norris Medical Library, University of Southern California, Los Angeles, CA; 2 aquezada@usc.edu, Supervisor, Access Services, Norris Medical Library, University of Southern California, Los Angeles, CA; 3 lezcano@usc.edu, Science and Engineering Librarian, Seaver Science Library, University of Southern California, Los Angeles, CA

## Abstract

**Objective:**

While studies from the early 1990s show that library staff in nonlibrarian roles interpret the term “paraprofessional” as being demeaning to their roles, no recent research has been conducted on this topic. This study aims to investigate if health sciences library staff continue to have similar negative associations with the term “paraprofessional” and to determine if another term is preferred.

**Methods:**

The authors conducted a literature review to identify terms used to categorize library staff in nonlibrarian roles. Using these terms, we created an online Qualtrics survey asking participants to rank terms by preference. We distributed the survey via thirty-six professional email discussion lists, including MEDLIB-L, thirty-three MLA chapter and caucus email discussion lists, DOCLINE-L, and ACRL-HSIG-L. Survey participants included full-time and part-time health sciences library staff in any nonlibrarian position. Responses from librarians were not accepted.

**Results:**

Based on 178 completed surveys, “library staff” was the top choice of 49% of participants, over “other” (19%), “paraprofessional” (13%), “library support staff” (11%), “paralibrarian” (7%), and “nonprofessional” (1%). Although “library staff” was the top choice of participants across all ages, older participants (aged 45–75) preferred “library support staff” and “paraprofessional” to a greater degree than younger participants (aged 18–44), while younger participants preferred “other” to a greater degree. Out of 36 participants who specifically mentioned the terms “paraprofessional” or “paralibrarian,” 32 (89%) of those comments were negative, indicating that the “para” in “paraprofessional” and “paralibrarian” is either insulting, inapplicable, or unfamiliar.

**Conclusions:**

Our results suggest that although the term “paraprofessional” may not intentionally be used to demean library staff, many library staff interpret the term to be demeaning to their roles. Instead, “library staff,” a more inclusive and less divisive term, was preferred by survey participants. In accordance with our results, we believe the term “paraprofessional” should no longer be used in library and information scholarly literature or professional discourse.

## INTRODUCTION

The definition of “paraprofessional personnel” in the *American Library Association (ALA) Glossary of Library & Information Science* reads:

A term used to designate *library staff* without professional *certification* who perform supportive duties, often at a high level, for professional personnel. The term is variously applied to personnel classified as *library associates* and *library technical assistants,* and, less precisely, to all members of the *support staff.* Synonymous with *paraprofessional* and *subprofessional personnel.* [1]

This definition highlights the fact that the term “paraprofessional” primarily refers to library personnel in nonlibrarian roles with high-level positions and responsibilities, but that the term is also used to refer to *all* members of the support staff [2]. These coexisting definitions are reflected in library literature. In 1992, Larry R. Oberg explained: “Paraprofessionals occupy a middle stratum of a three-tiered hierarchical staffing structure. Within this model, paraprofessionals are ranked below librarians, but above clerical workers” [3]. In 2012, Lihong Zhu stated, “In the previous five years, library paraprofessionals were still defined as library employees who did not have an MLIS [master's of library and information science]” [4]. The broader definition is reflected by the Library Support Staff Resource Center on the ALA website, which uses the term “library support staff” as a synonym for “paraprofessional” and lists a wide range of positions in this category from “library page” to “library information specialist” [5]. The website describes library support staff and paraprofessionals as being “involved in all library operations at all levels” with “the range and complexity of their duties vary[ing] with each position” [5].

Throughout this manuscript, the authors use “library staff” to refer to library personnel who are employed in nonlibrarian roles. We use “librarian” to refer to library personnel with MLIS or master's of library science (MLS) degrees who are employed in librarian positions.

There has been a significant amount of literature published on the expanding roles of library support staff and the blurring of lines between “professional” and “paraprofessional” roles and responsibilities [4, 6–9], but less research has been done on nomenclature and how library staff interpret or feel about the term “paraprofessional.” In the *Library Staff Development Handbook: How to Maximize Your Library's Most Important Resource*, Mary Grace Flaherty, AHIP, wrote:

The terms *library assistant* or *associate, paraprofessional, library technician* or *clerk, nonprofessional, support staff*, and *paralibrarian* are used somewhat interchangeably to describe positions in libraries that do not require a graduate degree. This is somewhat misleading, though, as there is such a wide range of positions that non-MLIS holders are occupying. Some manage libraries, some supervise staff; it is very difficult to create one category that could represent all library support staff. Additionally, some of the terms are reportedly found demeaning by some support staff members, especially *nonprofessional*, as it carries the implication of lack of professionalism on the part of support staff. [10]

Similarly, the ALA Library Support Staff Resource Center website states:

We have even seen the terms “non-professionals” and “sub-professionals.” While no consensus has yet formed on a name for this group, support staff are solidly against these last two labels. They do not reflect the dedication and talent workers bring to their jobs. [5]

In addition to negative feelings about the terms “nonprofessional” and “subprofessional,” research has indicated that some library support staff perceived the term “paraprofessional” to be demeaning as well. In focus groups conducted for the World Book ALA Goal Award Project on Library Support Staff in 1991 [11], many library support staff expressed a dislike for “paraprofessional,” “nonprofessional,” and other terms that place the role of library support staff in opposition to professionalism. The focus groups revealed that “for many paraprofessionals, professionalism is an attitude, and many feel that they competently demonstrate that attitude.” Thus, the use of the term “professionals” to distinguish librarians with an MLS from those library workers who did not have an MLS also presented difficulties. Many participants expressed that problems with terminology had a negative impact on staff morale due to feelings of being left out or demeaned. The focus groups highlighted that “terminology problems related to library staff reflect underlying issues,” and in libraries, “these issues stem from the shifting and blending of responsibilities within the field and in each library workplace.” One participant commented: “Paraprofessional makes it sound like you're only half a professional.” Overall, the focus groups revealed that “there is no satisfactory or agreed upon terminology for library staff who do not have a master's degree in library and information studies,” and participants felt that a consensus on a new term that was empowering was needed [11].

In 1992, Oberg surveyed academic library directors on the role and status of paraprofessional staff and included a question about nomenclature. Oberg wrote:

The responses of the library directors surveyed make it clear that the term paraprofessional is a highly charged one. A number of these directors responded that they simply do not like it. Others reported that it is considered demeaning by some staff who prefer such terms as *support professional* or even *librarian.* [3]

Intentional or not, the words that are used to refer to others in the workplace can carry implications of mutual respect or the lack thereof. Despite controversies about the term “paraprofessional” as shown in previous studies [3, 11], this term is used in library and information literature and by organizations, often in association websites and awards.

The aim of our study was to investigate term preferences for job roles of library personnel in nonlibrarian roles (henceforth, referred to as library staff). To do this, we conducted a survey of health sciences library staff to rank and comment on a list of commonly used terms. We hypothesized that a significant number of library staff would have negative associations with the term “paraprofessional.”

## METHODS

### Design and participants

We developed an anonymous ten-question survey in Qualtrics (Qualtrics, Provo, UT), designed to determine which terms library personnel in nonlibrarian roles preferred and how participants interpreted or perceived the terms. Two questions focused on preferred terms, and eight questions collected demographic information, including job title, job responsibilities, type of library, education level, age, racial or ethnic identity, and gender identification. Supplemental Appendix A provides a copy of the survey.

Eligible survey participants included full-time and part-time medical and health sciences library personnel in any nonlibrarian position. We incorporated a required question that used branching to terminate the survey for respondents who indicated they were employed as librarians. As a result of this branching question, librarians could not complete the survey. To identify master's or doctorate (PhD) level library employees in roles other than librarian (e.g., a bioinformatics specialist with a PhD in bioinformatics), we included questions on job title, job responsibilities, and education level. Surveys were included in the results if they answered the term preference-ranking question. Surveys that did not respond to this question were considered incomplete.

The first question asked participants to rank a list of terms in order of preference. The list of terms included “paraprofessional,” “paralibrarian,” “library staff,” “library support staff,” “nonprofessional,” and “other.” Library and information science literature and association websites were analyzed to develop a list of terms synonymous with “paraprofessional” [1, 4, 5, 10, 12]. The main criteria in determining which terms to include on the list were that the term functioned as an umbrella term that encompassed a wide variety of job titles and positions, similar to “paraprofessional.” Terms such as “library technician,” “library assistant,” and “library associate” that were also used as specific job titles were not included because we were concerned that participants would choose their own job title rather than one of the umbrella terms. We did not intend to include the terms “nonprofessional” and “subprofessional,” used synonymously with “paraprofessional” in the past, because of their obviously negative implications. However, in a design oversight, the term “nonprofessional” was included, and participants were asked to rank this term.

To avoid bias toward any particular term, we did not use the terms from the list in the ranking question elsewhere in the survey. Also, we did not explicitly state that we were interested in how participants viewed the term “paraprofessional.” The second question allowed participants to provide comments and thoughts about any of the terms on the list. The purpose of this question was to gather qualitative data to help us understand why people preferred or did not prefer the terms on the list.

We included the eight demographic information questions primarily because we were interested in getting an overall picture of the survey participants. We were also interested to see if there were statistically significant relationships between term preference and education level, age, gender, or racial or ethnic identity of participants. Questions on job title, job responsibilities, and education level would also help us identify master's or PhD level library employees in roles other than librarian (e.g., a bioinformatics specialist with a PhD in bioinformatics) and analyze these responses separately, if necessary.

### Recruitment

We sent out an email invitation with a link to the survey on October 15, 2018, to prospective participants via thirty-six professional email discussion lists: MEDLIB-L (Medical Library Association), DOCLINE-L (National Library of Medicine), ACRL-HSIG-L (Association of College & Research Libraries Health Sciences Interest Group), and thirty-three Medical Library Association chapter, section, and special interest group email discussion lists. We sent follow-up invitations in November and closed the survey on Friday, November 16, 2018.

We received exempt status and approval for the study from the Institutional Review Board (IRB) at the University of Southern California (USC) prior to distribution.

### Data analysis

We used Qualtrics Data to filter data of participants who answered the ranking question on term preference. We exported the data from Qualtrics and analyzed the data in Excel. We created tables to extract relevant data pertaining to the ranking question, qualitative comments, and participant characteristics. We used Qualtrics Reports to visualize the ranking question data and double-checked the visualization data against the raw data in Excel.

For qualitative responses, we used grounded theory to review the free-text data for repeated themes and coded these data into categories based on these themes. One author coded the themes initially, and two authors reviewed the themes for accuracy. All authors agreed on the themes.

We coded qualitative participant comments about the terms into four themes based on positive, neutral, or negative feelings about terms. For participants who expressed more than one theme in their response, each theme was identified and counted. Hence, the numbers did not reflect a one-to-one ratio. For example, if a participant expressed a dislike for “paraprofessional,” “paralibrarian,” and “nonprofessional,” this response would have been included in two separate themes.

Using guidance from a bioinformatics specialist to analyze the statistical relationship between term preference and education level, age, gender, and racial or ethnic identity of participants, we first used an online chi-square test calculator [13]. For significant relationships found by the chi-square test, we further probed detailed term preferences applying the *Z*-test for 2 population proportions [14]. We used *p*<0.05=significant for both the chi-square test and *Z*-test. Supplemental Appendix B details our methods for statistical significance.

## RESULTS

### Participant characteristics

There were a total of 286 survey attempts, including 108 incomplete attempts that were eliminated. Incomplete surveys included 58 attempts made by librarians and 50 attempts made by library staff in nonlibrarian roles who did not answer the ranking question. All survey results in this paper were based on 178 respondents who completed the term-preference-ranking question.

Participants reflected a wide variety of traditional library staff job titles, including library assistant (31%), specialist (12%), associate (9%), coordinator (8%), manager (8%), supervisor (4%), clerk (2%), administrative and office roles (5%), information technology (IT) roles (3%), and other miscellaneous job titles that fell under traditional library responsibilities (5%).

Most participants (98%) were employed at academic health sciences libraries and hospitals. Many participants had at least a bachelor's degree (49%), and many (23%) had a master's degree as well. The majority (67%) did not have a degree or certificate in library and/or information science, but 14% had an MLIS or MLS or were currently enrolled in a degree program (5%). There were no master's or PhD level library employees in roles other than librarian (e.g., a bioinformatics specialist with a PhD in bioinformatics).

Seventy-three percent of the participants identified as female, 24% as male, 1% as gender nonconforming, and 2% preferred not to answer. Sixty percent identified as white; 12% identified as Black or African American; 11% identified as Hispanic, Latino, or Spanish origin; 9% identified as Asian; 6% identified as mixed ethnicity or race; 2% identified as other; and 9% preferred not to answer. The majority fell within the 25–64 age range: 25% in the 25–34 range, 19% in the 35–44, 2% in the 18–24 range, 7% in the 65–74 range, and 0.5% in the 75+ range. Supplemental Appendix C presents participant characteristics data in table format.

Using the chi-square test, we found no statistically significant relationship between term preference and education level [χ^2^(8, n=176)=6.597, *p*=0.692], term preference and gender [χ^2^(4, n=171)=1.633, *p*=0.803], and term preference and racial or ethnic identity [χ^2^(4, n=161)=8.044, *p*=0.0900]. We found a statistically significant relationship between term preference and age [χ^2^(4, n=177)=12.055, *p*=0.0169]. Although participants across all ages overwhelmingly ranked “library staff” as the #1 choice, participants between the ages of 45 and 75 preferred “library support staff” to a greater degree than participants between the ages of 18 and 44 (*Z*=–2.5589, *p*=0.0105). Participants between the ages of 45 and 75 also marginally preferred “paraprofessional” to a greater degree than participants between the ages of 18 and 44 (*Z*=–1.6956, *p*=0.089). Participants between the ages of 18 and 44 marginally preferred “other” to a greater degree than participants between the ages of 45 and 75 (*Z*=1.9333, *p*=0.0536).

### Term preferences

The term-ranking question asked participants to rank a list of terms in order of preference from #1 to #6. The list of 6 terms included “paraprofessional,” “paralibrarian,” “library staff,” “library support staff,” “nonprofessional,” and “other.”

**Figure 1:**
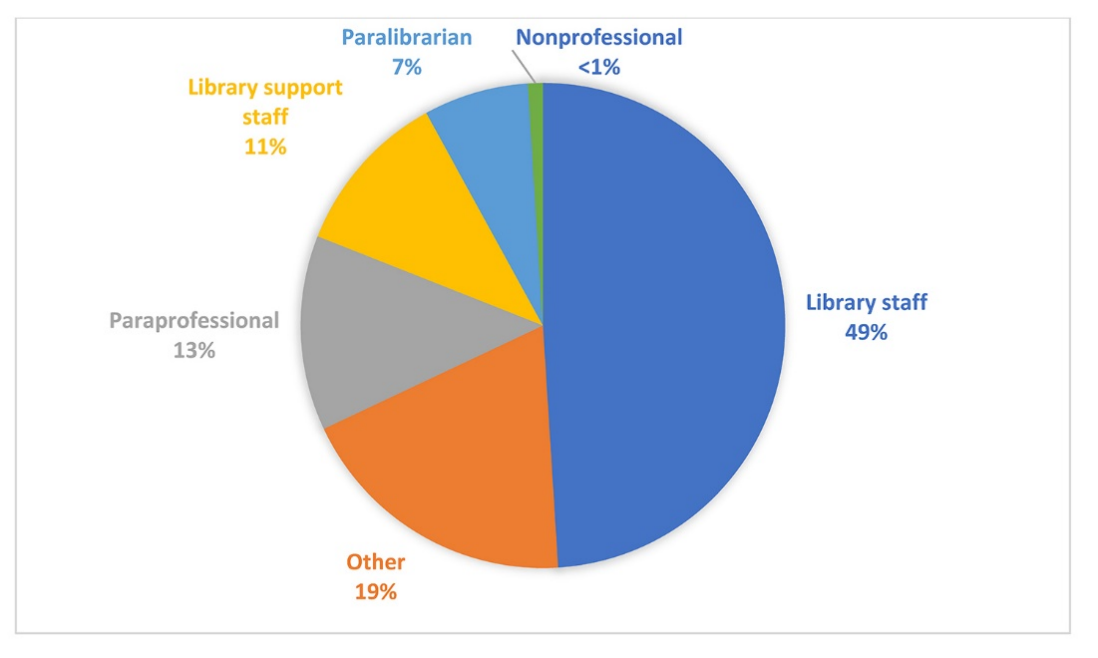
Participants' top choices for term preferences (n=178)

Figure 1 illustrates participants' choices for the #1 preferred term, meaning that participants chose these terms as their top choice. “Library staff” was the top choice for participants, with nearly half (49%, n=87) selecting this as their #1 preferred term. “Other” reflected the top choice of 19% of participants (n=34), with “paraprofessional,” “library support staff,” and “paralibrarian” representing between 7% and 13% of top choices. “Nonprofessional” received the least amount of top choices (<1%, n=1).

### Other suggested terms

Participants were provided the opportunity to fill in the blank for the “other” ranking, and fifty-nine participants made suggestions. Very few of the suggestions were broad umbrella terms that applied across the variety of positions and responsibilities that library staff have, and most reflected actual job titles that participants held, including “library assistant,” “library associate,” “library technician,” and “library specialist.” The suggestions that reflected broad umbrella terms included “librarian,” “non-degreed librarian,” and “professional staff,” suggesting that library staff were looking for more respect for their work. Supplemental Appendix C presents these data in table format.

### Qualitative comments

A total of ninety-five participants provided comments about the terms in the survey, and twenty-four comments were excluded from analysis, including eighteen in which the participant answered “no comment” or “N/A” and six that were deemed too ambiguous to fit into a theme. The seventy-one remaining comments were coded into themes. Table 1 lists direct quotes reflecting a selection of themes and the number of participants who expressed each theme. Supplemental Appendix D provides a complete list of comments and coding documentation.

**Table 1:** Comments (total comments coded n=71)

Theme	Direct quotes supporting theme	# Participants expressing the theme	
Expressed positive or neutral feelings about “paraprofessional” or “paralibrarian”	“Paralibrarians seems to be the best title for non-librarian staff because it does indicate our support of the library as well as indicates that many of us actually take on librarian responsibilities.”	4	(7%)
	“I refer to myself as a paraprofessional, although some of my duties include librarian work.”		
	“Never heard of the term paralibrarian. I like the term paraprofessional. I always thought of myself as library staff and not important as librarians.”		
Expressed negative feelings toward “para” prefix, “paraprofessional,” or “paralibrarian.” Expressed that the terms were degrading, strange, not applicable, or unfamiliar.	“Terms containing prefixes such as ‘para' and ‘non' are not preferable because they imply a lesser-than quality, a shortfalling.”	32	(45%)
	“Para something seems not very professional.”		
	“I find paraprofessional and nonprofessional insulting.”		
Expressed that the term “nonprofessional” is degrading, demeaning, or insulting and/or should be on the list at all.	“Nonprofessional sounds highly condescending. We are all expected to act professionally, therefore we are professionals, all of us. The ‘para' words annoy me less but they can definitely be used poorly in a certain context.”	46	(65%)
	“I hate ‘paraprofessional,' ‘paralibrarian,' and most certainly ‘NONprofessional.' Ugh. I want them to be not on this list.”		
	“Not sure why nonprofessional is listed?”		
Expressed a dislike for all or majority of terms but did not specifically state which terms	“They actually all suck except for ‘Library Staff'—which is what everyone in the library should be called. (I hold an MLIS).”	8	(11%)
	“These are all insulting and pointless. If you want to support the staff, provide opportunities for growth and advancement within the library.”		
	“I use ‘library staff' or ‘library support staff' only, not any of the other terms.”		

Of the 36 comments that specifically mentioned the “para” prefix, “paraprofessional,” and “paralibrarian,” 32 interpreted the term negatively. Participants who expressed a dislike for the term “paraprofessional” noted that the “para” implied being less than or not professional, and those who disliked the term “paralibrarian” expressed that this term was unfamiliar and confusing. Forty-six participants (65%) expressed that the term “nonprofessional” was insulting. Eight participants expressed a dislike for all or the majority of the terms listed, and some pointed to “library staff” and “library support staff” as the only preferred terms.

## DISCUSSION

### Term preference

“Library staff” was the overwhelmingly preferred term in our survey, with nearly half of participants selecting this as their top choice (Figure 1). The term “other” ranked second for participants' top preferred term, suggesting that for some, none of the terms on the list were acceptable as a top choice. Less than a quarter of participants chose “paraprofessional” or “library support staff” as a top choice, indicating far less support for either of these terms. Looking at participant comments, it was perhaps not surprising that “library support staff” and “paraprofessional” received similar rankings because the word “support” implied a hierarchy in a similar way that the “para” in paraprofessional did. For example, one participant noted: “Library Support Staff is a little better, but as with ‘nonprofessional' it creates a hierarchy where none may actually exist.”

In alignment with the previous research from the early 1990s on nomenclature, our results show that the term “paraprofessional” continues to be highly controversial. As so many of our survey comments demonstrated, the prefix “para” when juxtaposed with “professional” implied a lack of professionalism. For example, one participant commented: “Terms containing prefixes such as ‘para' and ‘non' are not preferable because they imply a lesser-than quality, a shortfalling.”

The word “professional” in the library environment can refer to the professional degree, MLIS or MLS, but it can also refer to professionalism. As one participant noted: “We are all expected to act professionally, therefore we are professionals, all of us.” Another participant stated: “[I] dislike the ‘para' approaches. I vehemently reject the idea that I am not a professional; I am simply a different kind of professional.” Together, these comments illustrate that library staff often think of themselves as professionals who hold professional positions and act professionally at work, even if their positions are different than librarians. In other words, there is room for more than one kind of professional in libraries.

In contrast to “paraprofessional,” “library staff” is a more inclusive term and can be used as an umbrella term to apply to all employees, including librarians and other personnel. The ALA Policy Manual outlines guidelines for inclusiveness and mutual respect: “Library employers that have developed respectful organizational cultures with inclusive language and developmental opportunities for all library workers should be recognized as models for others” [15]. In another ALA memo, guidelines for inclusive language are further discussed: “Unless greater specificity is necessary, the American Library Association uses inclusive language, such as: all library workers, all library staff, all library personnel, or librarians and support staff” [16].

In many instances, “library staff” (or similar broad designations, such as “library personnel” or “library employees”) can be used to address all staff in a variety of positions, including librarians and other staff. This does not mean that librarians, library assistants, library supervisors, library technicians, and others do not have different degrees, roles, and responsibilities (although roles can overlap at times), but it does suggest that different roles can all fall under the larger category of “library staff” or “library employee” rather than implying a hierarchy with the binary “professional” versus “paraprofessional.” In other instances, when there is a need to differentiate between librarians and personnel in nonlibrarian roles, inclusivity can be created by using preferred terms, such as librarians and staff or library faculty and staff, for example. Every library environment is unique, and inclusive language in each library may look a little different.

### Term preference and participant characteristics

Our findings demonstrated that “library staff” was the preferred term of participants, regardless of education level, age, gender, and racial or ethnic identity.

Although participants across all ages overwhelmingly ranked “library staff” as their top choice, older participants (aged 45–75) preferred “library support staff” and “paraprofessional” to a greater degree than younger participants (aged 18–44), while younger participants preferred “other” to a greater degree. The class of academic library worker categorized as “paraprofessional” emerged in the post–World War II period “as academic librarians busied themselves with their newfound faculty status requirements of teaching, research, and governance” and thus “became more and more dependent upon support staff” [3]. Because the term “paraprofessional” was adopted by librarians in this era and the terms “paraprofessional” and “support staff” are so connected, it was possible that older participants were more comfortable with these terms than their younger counterparts. This generational difference could also explain why younger participants preferred “other” to a greater degree than their older counterparts, suggesting that the younger generation was less familiar with or tolerant of terms like “paraprofessional” or “support staff.”

### Professional hierarchies

The problem with the term “paraprofessional” is that it implies a hierarchical binary between “professional” and “paraprofessional,” suggesting that those in the “paraprofessional” category do not practice professionalism. The results of this research bring to the surface professional hierarchies that exist in medical and health sciences libraries and the possible negative implications for librarian and library staff relationships due to the usage of divisive terms, such as “paraprofessional.” Other researchers have reported that professional divisions in libraries often result in tense and difficult relationships between librarians and library staff that can contribute to low workplace morale [17–19]. In 2018, Sarah Vela argued that professional hierarchies are one of the largest problems libraries face because “any degree of resentment or antagonism between groups can thus be very problematic, as it can prevent the transfer of knowledge from occurring” [17]. For example, a librarian's fear of loss of status or a library technician's resentment of not being recognized for contributions and skills can impede meaningful communication and “derail efforts to improve knowledge sharing in an organization” [17]. The sentiments expressed by the majority of participant comments in our survey indicate a perceived lack of value for the type of work library staff undertake. These negative implications can directly affect an organization's culture, working relationships, and library services.

The lack of diversity is a long-standing issue in the field, and there is a need for the structures and systems that maintain whiteness in librarianship to be examined and remedied [20]. Diversity initiatives in librarianship have failed to make a meaningful difference, and the number of librarians from racially and ethnically diverse backgrounds remains unacceptably low [21, 22].

Although the majority of both librarians and library staff are white women, staff are more racially and ethnically diverse, with up to 74.8% of library staff identifying as white, compared to up to 86.7% of librarians [23–27]. Our survey results reveal even more racial and ethnic diversity (60% white) than these existing statistics [23, 27]. In this context, the use of the word “paraprofessional” is even more problematic. In addition to implying hierarchical binaries and discounting staff roles, responsibilities, and expertise, the use of this term highlights the fact that systemic white privilege in institutions has long linked the notion of “professionalism” to whiteness [20].

In 2017, MLA announced the formation of the Diversity and Inclusion Task Force to “evaluate and improve MLA practices as they relate to diversity and inclusion” [28]. Diversity and inclusion initiatives are primarily centered on race, ethnicity, gender, and sexuality, but inclusivity is also applicable to professional roles. The more professional hierarchies are broken down, the more inclusive and welcoming libraries will be for both librarians and staff. Medical and health sciences libraries must commit to diversity, equity, and inclusion on multiple fronts, including discontinuing the use of divisive terms such as “paraprofessional” in efforts to create library cultures that respect librarians and library staff equally.

We hope that the results of this research will prompt others to discuss the role that language plays in contributing to inclusive library workplaces and organizations. For example, as a result of this survey, the Medical Library Group of Southern California and Arizona (MLGSCA) changed the name of the MLGSCA Paraprofessional of the Year Award to the MLGSCA Library Staff Excellence Award [29].

### Limitations

The oversight leading to the inclusion of the term “nonprofessional” on the list for term preference ranking was a limitation. By including the term on the list, participants were inadvertently insulted, which was counter to the purpose of the study. “Nonprofessional” is not an acceptable term to refer to any category of library employees. Although this term has been used in the past to refer to nonlibrarian employees, the term has already been established as unacceptable, and it should have been removed prior to survey distribution [5, 10].

To eliminate bias and avoid leading participants, we did not use the terms that participants were asked to rank anywhere else in the survey or email distribution language. However, this approach resulted in the use of noninclusive language, such as “nonlibrarian,” in our survey description and recruitment email communication. The term “nonlibrarian” defines employees by what they are not rather than what they are. A primary goal of our study was to develop more inclusive language and communication, and by utilizing the term “nonlibrarian,” we reinforced practices that we hoped to change.

One opportunity for further research would be to conduct a larger survey for all types of libraries rather than only health sciences libraries to see if the results are replicated in a larger sample in a broader library context. However, we believe that the results from our survey are applicable outside of health sciences libraries: any indication that library staff in any environment find the term “paraprofessional” insulting should be enough to stop using the word altogether in the field. On the other hand, there may be other terms that are more preferable than “library staff” that we did not identify or include in this survey.

## CONCLUSION

“Library staff” was the overwhelmingly preferred term of participants in our study, and many expressed that terms like “paraprofessional” and “library support staff” can create unnecessary professional hierarchies and diminish staff roles and contributions. Although the term “paraprofessional” may not intentionally be used to demean library staff, our findings show that many library staff interpret the term as demeaning to their roles. In accordance with these results, we believe the term “paraprofessional” should no longer be used in library and information scholarly literature or professional discourse. Words matter: if the intention is truly to create library environments where all employees are equally valued, the language used must reflect the inclusive library spaces.

## Data Availability

All data associated with our results is available via Open Science Framework at https://osf.io/ea328.

## SUPPLEMENTAL FILES

**Appendix A:** Terminology preferences of nonlibrarian medical and health sciences library personnel survey

**Appendix B:** Statistical significance tests

**Appendix C:** Results supplemental tables

**Appendix D:** Qualitative comments coding documentation
