# Supplementary material for: Words matter: interpretations and implications of “para” in paraprofessional
Source: J Med Libr Assoc. 2021 Jan 1;109(1):13–22. doi: 10.5195/jmla.2021.933 (PMC7772989; doi:10.5195/jmla.2021.933)
Supplement: Supplementary file 1 — Appendix A: Terminology preferences of nonlibrarian medical and health sciences library personnel survey [file jmla-109-1-13-s01.pdf]

JMLA Journal of the Medical Library Association

Supplemental content to
J Med Libr Assoc. Jan;109(1):dx.doi.org/10.5195/jmla.2021.933
www.jmla.mlanet.org
© Schilperoort, Quezada, Lezcano 2021

Words matter: interpretations and implications of "para" in paraprofessional

Hannah Schilperoort; Alvaro Quezada; Frances Lezcano

APPENDIX A

Terminology preferences of nonlibrarian medical and health sciences library personnel survey

Consent page

Survey Title: Terminology Preferences of Nonlibrarian Medical and Health Sciences Library Personnel

Principal investigators: Hannah Schilperoort, information services librarian (schilper@usc.edu, 213.821.3008), Alvaro Quezada, supervising library assistant (aquezada@usc.edu, 323.442.1122), and Frances Lezcano, access services manager (lezcano@usc.edu, 323.442.3313); Norris Medical Library, University of Southern California

You are invited to participate in an online survey designed to determine terminology preferences of nonlibrarian medical and health sciences library personnel.

Library personnel are almost universally divided into two categories: librarians and nonlibrarians. In most cases, librarians have master's degrees in library and/or information science, while nonlibrarians do not. Some nonlibrarians may have library technical degrees or certifications. This distinction is not always accurate because some library personnel who are employed as librarians may not have a master's degree in library and/or information science and some library personnel who are employed as nonlibrarians may have a master's degree in library and/or information science.

This survey is about terminology and the language that is used to define library personnel roles. The purpose of this study is to identify the preferred terminology that library personnel in nonlibrarian positions working in medical and health sciences libraries want to use to refer to themselves as a *collective group.*

Librarians are referred to as librarians or information professionals and library faculty in academic settings. However, there is no agreed upon terminology for nonlibrarian personnel. Library personnel in this group include library assistants, clerks, technicians, supervisors, aids, pages, and many other titles.

As a collective group, library personnel in this category have been referred to as library staff, support staff, paralibrarians, paraprofessionals, nonprofessional, and other terms. We hope this survey will result in getting closer to establishing a preferred overarching terminology for library personnel in nonlibrarian positions.

The survey consists of a mix of eight multiple choice and two open-ended questions written in English. This survey should take you less than ten minutes to complete.

There are no foreseeable physical or emotional risks associated with taking this survey. Potential benefits associated with this survey include the possibility of establishing a preferred term for the collective group of nonlibrarian library personnel.

Your responses will remain anonymous. Although the results of this survey may be published, no identifying information will be collected.

Your participation is voluntary. You have the right to not answer questions that you do not wish to answer. You are free to exit the survey at any time. Questions that you answered prior to submitting the survey will be recorded.

SC
Supplemental content to
J Med Libr Assoc. Jan;109(1):dx.doi.org/10.5195/jmla.2021.933
www.jmla.mlanet.org
© Schilperoort, Quezada, Lezcano 2021

Choosing to continue indicates that you:

1. Have read and understood the terms and conditions of the survey
2. Agree with the terms and conditions of the survey
3. Are you at least 18 years old

Would you like to continue?

- O Yes
- O No

[If Would you like to continue?=No]
Thank you for your interest.
[Skip To: End of Survey If Thank you for your interest.() Is Displayed]

[If Would you like to continue?=Yes]
Are you currently employed as a librarian?

- O Yes
- O No

[If Are you currently employed as a librarian?=Yes]
Thank you for your interest. We are only accepting responses from library personnel who are not employed as librarians.

[Skip To: End of Survey If Thank you for your interest. We are only accepting responses from library personnel who are not employed as a librarian () Is Displayed]

[If Are you currently employed as a librarian?=No]
How do you identify as part of a collective group differentiated from librarians? Drag and place the terms in order of preference, with #1 being your most preferred term and #10 your least.

Note: We want to know how you identify as part of a collective group (differentiated from librarians) rather than your individual job title.

______ Paraprofessional
______ Paralibrarian
______ Library staff
______ Library support staff
______ Nonprofessional
______ Other

Do you have any comments about any of the terms listed above?

____

JMLA Journal of the Medical Library Association

SC
Supplemental content to
J Med Libr Assoc. Jan;109(1):dx.doi.org/10.5195/jmla.2021.933
www.jmla.mlanet.org
© Schilperoort, Quezada, Lezcano 2021

What is your official individual job title?

____

What is your primary job responsibility? Choose all that apply.

- O Acquisitions
- O Administration
- O Cataloging
- O Circulation
- O Collections
- O Interlibrary loan
- O Information technology (IT)
- O Supervision
- O Other ____

At what type of library do you work? Choose all that apply.

- O Academic
- O Hospital
- O Corporate
- O Other ____

What is the highest level of education you have completed? If currently enrolled, highest degree received?

- O High school diploma
- O Some college credit, no degree
- O Trade/technical/vocational training
- O Associate's degree
- O Bachelor's degree
- O Master's degree
- O Doctoral degree
- O Postdoctoral degree

Do you have a certificate or degree in library and/or information science? If so, what is the title of your certificate or degree?

- O No
- O Yes ____
- O Currently enrolled ____

JMLA Journal of the Medical Library Association

SC
Supplemental content to
J Med Libr Assoc. Jan;109(1):dx.doi.org/10.5195/jmla.2021.933
www.jmla.mlanet.org
© Schilperoort, Quezada, Lezcano 2021

What is your age?

- ○ 18–24
- ○ 25–34
- ○ 35–44
- ○ 45–54
- ○ 55–64
- ○ 65–74
- ○ 75 or older

With which racial or ethnic groups do you most identify? Select all that apply.

- ○ American Indian or Alaska Native
- ○ Asian
- ○ Black or African American
- ○ Hispanic, Latino, or Spanish origin
- ○ Native American
- ○ Native Hawaiian/Pacific Islander
- ○ White
- ○ Other
- ○ Prefer not to answer

Which of the following best describes your gender identity?

- ○ Male
- ○ Female
- ○ Gender variant/Nonconforming
- ○ Prefer not to answer
