# Supplementary material for: Words matter: interpretations and implications of “para” in paraprofessional
Source: J Med Libr Assoc. 2021 Jan 1;109(1):13–22. doi: 10.5195/jmla.2021.933 (PMC7772989; doi:10.5195/jmla.2021.933)
Supplement: Supplementary file 2 — Appendix B: Statistical significance tests [file jmla-109-1-13-s02.pdf]

JMLA Journal of the Medical Library Association

SC
Supplemental content to
J Med Libr Assoc. Jan;109(1):dx.doi.org/10.5195/jmla.2021.933
www.jmla.mlanet.org
© Schilperoort, Quezada, Lezcano 2021

Words matter: interpretations and implications of “para” in paraprofessional

Hannah Schilperoort; Alvaro Quezada; Frances Lezcano

APPENDIX B

Statistical significance tests

Statistical significance: term preference and patient characteristics

We first used an online chi-square test calculator (https://www.socscistatistics.com/tests/chisquare2/default2.aspx) to analyze the statistical relationship between term preference and education level, age, gender, and race and ethnicity of participants. The numbers in the chi-square test tables represent the number of participants in each category who chose the term as their #1 choice.

For significant relationships found by the chi-square test, we further probed detailed term preferences applying the *Z*-test for two population proportions (https://www.socscistatistics.com/tests/ztest/default2.aspx).

For both chi-square test and *Z*-test, we used $p<0.05$=significant.

We consulted a bioinformatics specialist for guidance on calculating and analyzing statistical significance.

Gender: chi-square test

	Male	Female	Row totals
Library staff	24	61	85
Library support staff	5	15	20
Paraprofessional	4	18	22
Paralibrarian	2	10	12
Other	7	25	32
Column totals	42	129	171 (grand total)

Did not include: Prefer not to answer=2, Gender nonconforming=2, and Nonprofessional=1 because numbers were too low to statistically analyze.

$\chi^2(4, n=171)=1.633$, $p=0.803$.

The result is not significant at $p<0.05$.

SC
Supplemental content to
J Med Libr Assoc. Jan;109(1):dx.doi.org/10.5195/jmla.2021.933
www.jmla.mlanet.org
© Schilperoort, Quezada, Lezcano 2021

Education level: chi-square test

	Associate's degree and below	Bachelor's degree	Master's degree and above	Row totals
Library staff	17	45	24	86
Library support staff	8	8	4	20
Paraprofessional	7	12	4	23
Paralibrarian	3	6	4	13
Other	11	16	7	34
Column totals	46	87	43	176 (grand total)

Did not include: No answer=1 and Nonprofessional=1 because numbers were too low to statistically analyze. Combined education levels into broader categories because not enough numbers were in each category for statistical analysis.

χ^2(8, n=176)=6.597, *p*=0.692.

The result is not significant at $p<0.05$.

Race/ethnic identity: chi-square test

	People of Color (POC)/Non-white: Asian: 9; Black/African American: 22; Hispanic/Latino/Spanish origin: 11; Multiracial: 10; Other: 3	White	Row totals
Library staff	24	56	80
Library support staff	4	14	18
Paraprofessional	12	8	20
Paralibrarian	5	7	12
Other	10	21	31
Column totals	55	106	161 (grand total)

Did not include: Prefer not to answer=16. Did not include: Nonprofessional=1 because numbers were too low to statistically analyze. Combined POC identities into 1 large category because there were not enough numbers in each category for statistical analysis.

χ^2(4, n=161)=8.044, *p*=0.0900.

The result is not significant at $p<0.05$.

JMLA Journal of the Medical Library Association

SC
Supplemental content to
J Med Libr Assoc. Jan;109(1):dx.doi.org/10.5195/jmla.2021.933
www.jmla.mlanet.org
© Schilperoort, Quezada, Lezcano 2021

Age range: chi-square test

	18–44	45–74	Row totals
Library staff	44	43	87
Library support staff	4	16	20
Paraprofessional	7	16	23
Paralibrarian	7	6	13
Other	21	13	34
Column totals	83	94	177 (grand total)

Did not include: Nonprofessional=1 because number was too low to statistically analyze. Combined age ranges because there were not enough numbers in each category for statistical analysis.

$\chi^2(4, n=177)=12.055$, $p=0.0169$.

The result is significant $p<0.05$.

Age range: Z-test

	18–44	45–74	*p*-value for z test for 3 population proportions
Library staff	0.530120482	0.457446809	0.33204
Library support staff	0.048192771	0.170212766	0.01046=significant
Paraprofessional	0.084337349	0.170212766	0.0891=trends toward significance
Paralibrarian	0.084337349	0.063829787	0.60306
Other	0.253012048	0.138297872	0.0536=very borderline significant

Participants between the ages of 45 and 75 preferred “library support staff” to a greater degree than participants between the ages of 18–44 ($Z=-2.5589$, $p=0.0105$).

Participants between the ages of 45 and 75 also marginally preferred “paraprofessional” to a greater degree than participants between the ages of 18 and 44 ($Z=-1.6956$, $p=0.089$).

Participants between the ages of 18 and 44 marginally preferred “other” to a greater degree than participants between the ages of 45–75 ($Z=1.9333$, $p=0.0536$).
