# Supplementary material for: Words matter: interpretations and implications of “para” in paraprofessional
Source: J Med Libr Assoc. 2021 Jan 1;109(1):13–22. doi: 10.5195/jmla.2021.933 (PMC7772989; doi:10.5195/jmla.2021.933)
Supplement: Supplementary file 3 — Appendix C: Results supplemental tables [file jmla-109-1-13-s03.pdf]

JMLA Journal of the Medical Library Association

SC
Supplemental content to
J Med Libr Assoc. Jan;109(1):dx.doi.org/10.5195/jmla.2021.933
www.jmla.mlanet.org
© Schilperoort, Quezada, Lezcano 2021

Words matter: interpretations and implications of "para" in paraprofessional

Hannah Schilperoort; Alvaro Quezada; Frances Lezcano

APPENDIX C
Results supplemental tables

Participant characteristics (n=178)

Characteristic	n	%*
Job title		
Manager	15	8%
Supervisor	7	4%
Specialist	21	12%
Assistant	55	31%
Technician	19	11%
Associate	16	9%
Coordinator	14	8%
Clerk	3	2%
Administrative and office	9	5%
Information technology (IT)	6	3%
Other titles: responsibilities fall under traditional library titles and responsibilities	9	5%
No answer	4	2%
Type of health sciences library		
Academic	103	58%
Academic and corporate	1	0.5%
Hospital	41	23%
Academic and hospital	30	17%
Corporate	1	0.5%
Other (association)	1	0.5%
No answer	1	0.5%
Education		
High school diploma	5	3%
Some college credit, no degree	24	13%
Associate's degree	13	7%
Bachelor's degree	87	49%
Master's degree	41	23%
Doctorate degree	3	2%

SC
Supplemental content to
J Med Libr Assoc. Jan;109(1):dx.doi.org/10.5195/jmla.2021.933
www.jmla.mlanet.org
© Schilperoort, Quezada, Lezcano 2021

Characteristic	n	%*
Trade, technical, or vocational training	4	2%
No answer	1	0.5%
Degree or certificate in library and/or information science		
No	121	67%
Currently enrolled: master's	9	5%
Currently enrolled: certificate, associate's, and bachelor's	2	1%
Currently enrolled: did not state	3	2%
Yes: master's	25	14%
Yes: certificate, associate's, and bachelor's	14	8%
Yes: did not state	3	2%
Age		
18–24	4	2%
25–34	45	25%
35–44	34	19%
45–54	44	25%
55–64	37	21%
65–74	13	7%
75+	1	0.5%
Gender		
Female	130	73%
Male	42	24%
Nonconforming	2	1%
Prefer not to answer	4	2%
Race and ethnicity		
American Indian or Alaska Native, Asian, Native American, white	1	0.5%
American Indian or Alaska Native, Black or African American, Native American	1	0.5%
Asian	9	5%
Asian, Black or African American, white, Other	1	0.5%
Asian, Hispanic, Latino, or Spanish origin, Native American, white	1	0.5%
Asian, white	1	0.5%
Black or African American	22	12%
Black or African American, Native American, white	1	0.5%
Black or African American, Other	1	0.5%
Hispanic, Latino, or Spanish origin	11	6%
Hispanic, Latino, or Spanish origin, white	1	0.5%
Native American, white	1	0.5%

JMLA Journal of the Medical Library Association

Supplemental content to
J Med Libr Assoc. Jan;109(1):dx.doi.org/10.5195/jmla.2021.933
www.jmla.mlanet.org
© Schilperoort, Quezada, Lezcano 2021

Characteristic	n	%*
White	107	60%
White, Other	1	0.5%
Other	3	2%
Prefer not to answer	16	9%

* Rounded to the closest whole number.

Coding: participant characteristics

Manager

1. Circulation Manager
2. Library Manager
3. Library Manager 1
4. Access Services Manager
5. Library Operations Manager
6. Manager, Library Services—Reference and Education
7. library manager
8. Manager
9. Special Projects & Events Manager
10. Manager, User Experience Operations
11. Library Resources Manager
12. Library Circulation Manager
13. Circulation Manager
14. Resource Library Manager
15. Public Services and 3D Print Shop Services Manager, Work-Study Supervisor

Supervisor

1. library supervisor
2. Senior Library Supervisor
3. Supervisor
4. Supervisor, Library Information Resources
5. Library Supervisor I
6. Circulation Assistant Supervisor
7. Interlibrary Loan Supervisor

Specialist

1. Library Specialist
2. Library Services Specialist
3. Information Resources and Services Specialist
4. Library Specialist
5. Library Specialist
6. Library Information Specialist 3
7. Library Specialist
8. library services specialist
9. Electronic Journals Specialist
10. Library Specialist

Supplemental content to
J Med Libr Assoc. Jan;109(1):dx.doi.org/10.5195/jmla.2021.933
www.jmla.mlanet.org
© Schilperoort, Quezada, Lezcano 2021

11. Library Specialist
12. Library Specialist
13. Collections Specialist
14. Library Specialist; Operations & Service Point
15. Interlibrary loan specialist
16. Library Technical Specialist
17. Library Specialist I
18. Library Specialist
19. Library Specialist
20. Library Specialist
21. Multimedia Specialist

Assistant

1. Library Assistant
2. Library Assistant I
3. Library Assistant
4. library assistant
5. Library Assistant
6. Library Assistant (Supervisor)
7. Library assistant
8. Library Assistant IV
9. bibliographic assistant 1
10. Bibliographic Assistant
11. Library Assistant IV
12. Library Technical Assistant II
13. Interlibrary Loan Aisstant
14. Access Service Assistant
15. Library Assistant
16. Library Assistant IV
17. Technical Assistant
18. Library Assistant 2
19. Library Technical Assistant/Educational Coordinator
20. Senior Library Assistant
21. Library assistant
22. LIBRARY TECHNICAL ASSISTANT
23. LAII
24. Library Assistant
25. Patron Services Assistant
26. Library Assistant
27. Senior Library Assistant
28. Library Assistant IV
29. Library Assistant
30. Library Assistant
31. Library Asst ll
32. Library Research Assistant
33. Library Assistant
34. Library Assistant
35. Library Assistant
36. Librican Assistant
37. Library Assistant

SC
Supplemental content to
J Med Libr Assoc. Jan;109(1):dx.doi.org/10.5195/jmla.2021.933
www.jmla.mlanet.org
© Schilperoort, Quezada, Lezcano 2021

38. Library Assistant (Work Study)
39. Library Assistant
40. Library Assistant
41. Library Assistant
42. Library Assistant 3
43. Library Assistant
44. PAS/Reference Library Assistant
45. Technical Services assistant
46. Library Assistant
47. Library Assistant
48. Circulation Assistant
49. LIbrary Assistant
50. Library Assistant
51. Library Assistant
52. Library Assistant
53. Sr. Library Technical Assistant
54. Patron Services Assistant
55. "Special Collections Assistant"

Technician

1. Library Technician
2. Library Support Technician
3. University Library Technician
4. University Library Technician—Advanced
5. Library technician but I have Serials Specialist on my business cards
6. Library Technician II
7. Documentation technician
8. Library Technician
9. Library Technician Intermediate
10. library technician III
11. Library Technician
12. Library Technician 2
13. Library Technician
14. Libvrary Technician
15. Library Technician
16. Library Technician
17. Library Technician
18. library technician lead
19. Library Technician

Associate

1. Library Associate I
2. Library Information Associate
3. Library associate
4. Library Associate
5. Library Media Tech Associate
6. library associate
7. Sr. Library Associate I
8. library associate

JMLA Journal of the Medical Library Association

SC
Supplemental content to
J Med Libr Assoc. Jan;109(1):dx.doi.org/10.5195/jmla.2021.933
www.jmla.mlanet.org
© Schilperoort, Quezada, Lezcano 2021

9. Library Associate (Technical Services Manager)
10. Library Associate
11. Circulation Associate
12. Library Associate
13. Library Associate
14. Library Associate
15. I work at two libraries. Library Associate and Library Assistant
16. Operations Associate

Coordinator

1. [Interlibrary loan] ILL Lending Coordinator
2. Health Sciences Library Coordinator/Instructional Support Associate
3. System Coordinator (Prior it was Business Support Coordinator & before that Library Assistant)
4. Project Coordinator
5. Coordinator, Access Services and Educational Technology Resources
6. Coordindator
7. Administrative Coordinator
8. Public Services Coordinator
9. User Services Coordinator
10. Outreach Coordinator
11. Coordinator of the Medical Collection
12. CME Coordinator
13. Coordinator of Access Services
14. Information Services Cordinator

Clerk

1. Library Clerk
2. Senior Library Clerk
3. Library Clerk

Administrative and Office

1. Executive Assisatnt to the Chair
2. Administrative Associate
3. Administrative Coordinator
4. Clerical Office Coordinator
5. Office Manager
6. Project Manager
7. Program Specialist/Educational Consultant
8. Assistant director
9. Project Operations Specialist

IT

1. Library Database Specialist
2. Solutions Developer
3. Technologist
4. Senior Computing Specialist
5. Library Systems Specialist
6. Desktop Support Technician

SC
Supplemental content to
J Med Libr Assoc. Jan;109(1):dx.doi.org/10.5195/jmla.2021.933
www.jmla.mlanet.org
© Schilperoort, Quezada, Lezcano 2021

Other

1. Library Operations Administrator
2. Research Project Manager
3. Head of Access Services
4. Administrative Analyst
5. Digital Strategist
6. Medical Librarian
7. Health Educator
8. Library Desk Tech
9. Fiscal Specialist II

Terms suggested by participants (n=59)

Theme	Suggested terms that express the theme	# participants	
Terms that include the word librarian and titles usually reserved for librarians	Librarian, Student Librarian, Non-Degree Librarian, Professional, Information Professional, Non-Faculty Professionals, Information Specialist, Professional Staff	9	15%
Common job titles for library staff	Library Assistant, Library Associate, Library Technician, Library Specialist	38	64%
Denotes project coordination or project management	Library Coordinator, Research Project Manager	4	7%
Denotes administrative and office staff	Office manager, Admin Staff	4	7%
Miscellaneous	Nonlibrarian, Staff	4	7%

Coding: terms suggested by participants

Terms that include the word librarian and titles usually reserved for librarians

1. Librarian
2. Professional
3. Student librarian
4. non-degree Librarian
5. Information Professional
6. Information Professional
7. Professional Staff
8. Non-faculty professionals
9. Information Specialist

Common job titles for library staff

1. library assistant
2. Library Assistant
3. Library Technical Assistant
4. Library Associates
5. Library associates
6. Library Associate
7. Library Specialist
8. Library Specialist

JMLA Journal of the Medical Library Association

SC
Supplemental content to
J Med Libr Assoc. Jan;109(1):dx.doi.org/10.5195/jmla.2021.933
www.jmla.mlanet.org
© Schilperoort, Quezada, Lezcano 2021

9. Library Assistant
10. Library Assistant
11. Library Technician
12. Library Technical Assistant
13. Library Assistant
14. Library Assistant
15. Library Specialist
16. Library Specialist
17. Library Technician
18. Technician
19. Library Assistant
20. Library Specialist
21. library associate
22. Library Assistant
23. Library Technician
24. Library specialist
25. Library Technician
26. Library Technician
27. Library Technician
28. Library Supervisor
29. Library Assistant
30. Library Technician
31. Library Specialist
32. LibraryTechnical Staff
33. Library Specialist
34. Library Assistant
35. Technical specialist
36. Documentation technician
37. Circulation Staff
38. Library Media Tech Associate

Denotes project coordination or project management

1. Project Coordinators
2. Library Coordinator
3. Coordinator of the health collection
4. Research Project Manager

Denotes administrative and office staff

1. Office Manager
2. Office Coordinator
3. Admin Staff
4. Library Systems Admin

Miscellaneous

1. Anything but non-professional
2. Nonlibrarian
3. Staff
4. no term
