# Supplementary material for: Words matter: interpretations and implications of “para” in paraprofessional
Source: J Med Libr Assoc. 2021 Jan 1;109(1):13–22. doi: 10.5195/jmla.2021.933 (PMC7772989; doi:10.5195/jmla.2021.933)
Supplement: Supplementary file 4 — Appendix D: Qualitative comments coding documentation [file jmla-109-1-13-s04.pdf]

JMLA Journal of the Medical Library Association

Supplemental content to
J Med Libr Assoc. Jan;109(1):dx.doi.org/10.5195/jmla.2021.933
www.jmla.mlanet.org
© Schilperoort, Quezada, Lezcano 2021

Words matter: interpretations and implications of "para" in paraprofessional

Hannah Schilperoort; Alvaro Quezada; Frances Lezcano

APPENDIX D
Qualitative comments coding documentation

Coding for Table 2: qualitative comments

Survey question: Do you have any comments about any of the terms listed above?

All comments.

1. They are describe what I am not. None of them describe what I am.
2. they actually all suck except for "Library Staff"—which is what everyone in the library should be called. (I hold an MLIS).
3. These are all insulting and pointless. If you want to support the staff, provide opportunities for growth and advancement within the library.
4. There is nothing for administrative positions.
5. The term "nonprofessional" is insulting on a visceral level, though I understand why it might be used to differentiate between those with an MLIS and those without. "Library Support Staff" is a little better, but as with "nonprofessional" it creates a hierarchy where none may actually exist.
6. the term 'nonprofessional' deminishes the function. Just because someone has a role as library support staff, doesn't mean that they act notprofessionally.
7. The "para" prefix is degrading enough, but the inclusion of the term "nonprofessional" is egregiously insulting—I'm absolutely a professional! Do people really use that term in practice?!
8. terms don't give confidence to patrons, they often want to speak to a librarian as they expect them to be more knowledgeable even if it is a simple question.
9. Terms containing prefixes such as "para" and "non" are not preferable because they imply a lesser-than quality, a shortfalling.
10. Paraprofessionals usually have a certificate in Library Science.
11. Paraprofessional, paralibrarian, and nonprofessional do not apply to me.
12. Paraprofessional and Paralibrarian are not titles commonly known in a library setting and reductive to our goals.
13. Paraprofessional and nonprofessional signify nothing to me in the library context. They don't belong on the list.
14. Paralibrarians seems to be the best title for non-librarian staff because it does indicate our support of the library as well as indicates that many of us actually take on librarian responsibilities.
15. Paralibrarian sounds like an illness.
16. Paralibrarian seems like a hard title to explain to others, and non-professional sounds defeated.
17. Paralibrarian it's not used in Canada.
18. Paralibrarian isn't really a good way of describing support staff.
19. Para something seems not very professional.

JMLA Journal of the Medical Library Association
SC
Supplemental content to
J Med Libr Assoc. Jan;109(1):dx.doi.org/10.5195/jmla.2021.933
www.jmla.mlanet.org
© Schilperoort, Quezada, Lezcano 2021

20. Only two are used in our library. None of the other terms that differentiate us from each other mean anything.
21. only the first two are relevant.
22. Only identify with 1 and 2.
23. Of the options above, I would consider "nonprofessional" the least appropriate or relevant designation.
24. Not sure why nonprofessional is listed?
25. Nonprofessional sounds somewhat derogatory, as if referring to conduct.
26. Nonprofessional sounds demeaning.
27. Nonprofessional should be removed because Library Support Staff is already containing the meaning of nonprofessional.
28. Nonprofessional may have a negative connotation and imply the individual does not do important work.
29. Nonprofessional is way too negative to be used.
30. Nonprofessional is off-putting. I've never heard the term, "paralibrarian."
31. Nonprofessional is not good.
32. Nonprofessional is not a good term as it denotes inferior value and Parlibrarian confuses the distinction between Librarians and non-librarians. Paraprofessional is too generic.
33. Nonprofessional implies negativity.
34. Nonprofessional has terrible implications.
35. Nonprofessional has a very negative connotation and does not really signify anything.
36. Nonprofessional has a negative connotation, in my opinion.
37. Nonprofessional gives a negative connotation.
38. none.
39. Non-professional sounds like unprofessional—and I am very professional, and the para words sounds like I am somehow disabled.
40. Non professional needs to come off.
41. No comments.
42. No.
43. No.
44. no.
45. no.
46. No.
47. no.
48. no.
49. No.
50. No.
51. no.
52. no.
53. no.
54. no.

JMLA Journal of the Medical Library Association

SC
Supplemental content to
J Med Libr Assoc. Jan;109(1):dx.doi.org/10.5195/jmla.2021.933
www.jmla.mlanet.org
© Schilperoort, Quezada, Lezcano 2021

55. Never heard of the term paralibrarian. I like the term paraprofessional. I always thought of myself as library staff and not important as librarians.
56. Never heard of paralibrarian.
57. N/A.
58. Library staff.
59. I've never heard of "paralibrarian" or "paraprofessional." I would not like to be referred to in either of those ways.
60. I've never heard 'paralibrarian' before.
61. I'm used to para for paralegal but paralibrarian seems very strange to me.
62. I'd find "Nonprofessional" insulting, everyone on our team is "Library Staff." "Paralibrarian" sounds strange to me.
63. I would never use the terms Paralibrarian, Paraprofessional, or nonproprofessional to describe the group of non-librarians.
64. I use "library staff" or "library support staff" only, not any of the other terms.
65. I think the terms Paraprofessional, and Nonprofessional are demeaning!
66. I strongly dislike "nonprofessional."
67. I see myself as Library Staff only.
68. I refer to myself as a paraprofessional, although some of my duties include librarian work.
69. I really don't like "Nonprofessional" I am very knowlegable but did not have the option of going to school. Para…sounds too much like paratroopers and parachutes.
70. I really dislike the term "nonprofessional". It's too similar to "unprofessional."
71. I really dislike nonprofessional—it sounds dismissive of my role.
72. I prefer to think of us as a distinct group, and thus dislike the 'para' approaches. I vehemently reject the idea that I am not a professional; I am simply a different kind of professional.
73. I have worked in Medical Libraries for 26 years.
74. I have never heard the term Paralibrarian before and I would be offended if someone called me a nonprofessional.
75. I have never heard "Nonprofessional" and think that sounds a bit harsh/bad.
76. I have a Masters in Health Administration. I have worked in at a medical school for over thirty years. Why can I not move from Library staff?
77. I hate "paraprofessional", "paralibrarian", and most certainly "NONprofessional". Ugh. I want them to be not on this list.
78. I find the term "nonprofessional" suggests that I can't do the job and I dislike it.
79. I find paraprofessional and nonprofessional insulting.
80. I find most of the terms above to be very vague and applicable to many diffferent types of library staff roles.
81. I feel that non-professional and paraprofessional are semi insulting to those who are working full time in a professional office.
82. I feel that most people don't know what "paralibrarian" means.
83. I feel nonprofessional is completely unacceptable.
84. I don't feel as though I associate myself with non-professionals or even paraprofessionals.
85. I do not like the term Nonprofessional. It makes us sound "less than."

JMLA Journal of the Medical Library Association
Supplemental content to
J Med Libr Assoc. Jan;109(1):dx.doi.org/10.5195/jmla.2021.933
www.jmla.mlanet.org
© Schilperoort, Quezada, Lezcano 2021

86. I dislike the term Nonprofessional, I'm a professional with or without a master's degree.
87. I dislike all but the first one.
88. I believe some librarians treat us as if we are nonprofessional. I am not a fan of this term especially since we are usually the first people patrons approach for reference and technical help. I also get frustrated when we are asked to learn a new tool (e.g. database, citation manager) without much direction or a heads up. In other words there are a lot of decisions made without our input or any thought given to how it will impact our day to day job tasks.
89. I am almost always called a "librarian" by our patrons and people who work outside of the library world. I always identify myself as "library staff" and not a librarian because I do not have an MLS, but pretty much everyone says "You're a librarian. You do all the same things."
90. I added Project Coordinator not just because it is my job title. I am in a big institution, and I work frequently with Project Coordinators at departments outside of the library.
91. Both Para-s are troublesome, and nonprofessional is worst of all. We had an Ivy League PhD who would've been a paraprofessional.
92. Being part library staff is a professional position, so I feel the term "nonprofessional" is inappropriate.
93. "Nonprofessional" sounds highly condescending. We are all expected to act professionally, therefore we are professionals, all of us. The "para" words annoy me less but they can definitely be used poorly in a certain context.
94. "Nonprofessional" is insulting and never acceptable.
95. 'Nonprofessional' should not be listed. That is a terrible job title.

Positive or neutral feelings about paraprofessional or paralibrarian.

1. Paraprofessionals usually have a certificate in Library Science.
2. Paralibrarians seems to be the best title for non-librarian staff because it does indicate our support of the library as well as indicates that many of us actually take on librarian responsibilities.
3. I refer to myself as a paraprofessional, although some of my duties include librarian work.
4. Never heard of the term paralibrarian. I like the term paraprofessional. I always thought of myself as library staff and not important as librarians.

Expressed negative feelings toward "Para" prefix, paraprofessional, or paralibrarian. Expressed that the terms are degrading, strange, not applicable, or unfamiliar.

1. The "para" prefix is degrading enough, but the inclusion of the term "nonprofessional" is egregiously insulting—I'm absolutely a professional! Do people really use that term in practice?!
2. Terms containing prefixes such as "para" and "non" are not preferable because they imply a lesser-than quality, a shortfalling.
3. Paraprofessional, paralibrarian, and nonprofessional do not apply to me.
4. Paraprofessional and nonprofessional signify nothing to me in the library context. They don't belong on the list.
5. Paraprofessional and Paralibrarian are not titles commonly known in a library setting and reductive to our goals.
6. Paralibrarian sounds like an illness.
7. Paralibrarian seems like a hard title to explain to others, and non-professional sounds defeated.
8. Paralibrarian it's not used in Canada.
9. Paralibrarian isn't really a good way of describing support staff.

JMLA Journal of the Medical Library Association

Supplemental content to
J Med Libr Assoc. Jan;109(1):dx.doi.org/10.5195/jmla.2021.933
www.jmla.mlanet.org
© Schilperoort, Quezada, Lezcano 2021

10. Para something seems not very professional.
11. Nonprofessional is off-putting. I've never heard the term, "paralibrarian."
12. Nonprofessional is not a good term as it denotes inferior value and Parlibrarian confuses the distinction between Librarians and non-librarians. Paraprofessional is too generic.
13. Non-professional sounds like unprofessional—and I am very professional, and the para words sounds like I am somehow disabled.
14. Never heard of paralibrarian.
15. I've never heard of "paralibrarian" or "paraprofessional." I would not like to be referred to in either of those ways.
16. I've never heard 'paralibrarian' before.
17. I'm used to para for paralegal but paralibrarian seems very strange to me.
18. I'd find "Nonprofessional" insulting, everyone on our team is "Library Staff." "Paralibrarian" sounds strange to me.
19. I would never use the terms Paralibrarian, Paraprofessional, or nonproprofessional to describe the group of non-librarians.
20. I think the terms Paraprofessional, and Nonprofessional are demeaning!
21. I really don't like "Nonprofessional" I am very knowledgeable but did not have the option of going to school. Para…sounds too much like paratroopers and parachutes.
22. I prefer to think of us as a distinct group, and thus dislike the 'para' approaches. I vehemently reject the idea that I am not a professional; I am simply a different kind of professional.
23. I have never heard the term Paralibrarian before and I would be offended if someone called me a nonprofessional.
24. I hate "paraprofessional", "paralibrarian", and most certainly "NONprofessional". Ugh. I want them to be not on this list.
25. I find paraprofessional and nonprofessional insulting.
26. I feel that non-professional and paraprofessional are semi insulting to those who are working full time in a professional office.
27. I feel that most people don't know what "paralibrarian" means.
28. I don't feel as though I associate myself with non-professionals or even paraprofessionals.
29. I am almost always called a "librarian" by our patrons and people who work outside of the library world. I always identify myself as "library staff" and not a librarian because I do not have an MLS, but pretty much everyone says "You're a librarian. You do all the same things."
30. I am a professional with over 20 years experience in the high tech world which I also use in my role. I have an MLIS degree but by title is Coordinator. I use the skills that I have learnt in high tech daily so I find it insulting that I am referred to as a paraprofessional.
31. Both Para-s are troublesome, and nonprofessional is worst of all. We had an Ivy League PhD who would've been a paraprofessional.
32. "Nonprofessional" sounds highly condescending. We are all expected to act professionally, therefore we are professionals, all of us. The "para" words annoy me less but they can definitely be used poorly in a certain context.

JMLA Journal of the Medical Library Association

SC
Supplemental content to
J Med Libr Assoc. Jan;109(1):dx.doi.org/10.5195/jmla.2021.933
www.jmla.mlanet.org
© Schilperoort, Quezada, Lezcano 2021

Expressed that the term nonprofessional is degrading, demeaning, or insulting and/or should not be on the list at all.

1. The term "nonprofessional" is insulting on a visceral level, though I understand why it might be used to differentiate between those with an MLIS and those without. "Library Support Staff" is a little better, but as with "nonprofessional" it creates a hierarchy where none may actually exist.
2. the term 'nonprofessional' deminishes the function. Just because someone has a role as library support staff, doesn't mean that they act notprofessionally.
3. The "para" prefix is degrading enough, but the inclusion of the term "nonprofessional" is egregiously insulting—I'm absolutely a professional! Do people really use that term in practice?!
4. Terms containing prefixes such as "para" and "non" are not preferable because they imply a lesser-than quality, a shortfalling.
5. Paraprofessional, paralibrarian, and nonprofessional do not apply to me.
6. Paraprofessional and nonprofessional signify nothing to me in the library context. They don't belong on the list.
7. Paralibrarian seems like a hard title to explain to others, and non-professional sounds defeated.
8. Of the options above, I would consider "nonprofessional" the least appropriate or relevant designation.
9. Not sure why nonprofessional is listed?
10. Nonprofessional sounds somewhat derogatory, as if referring to conduct.
11. Nonprofessional sounds demeaning.
12. Nonprofessional should be removed because Library Support Staff is already containing the meaning of nonprofessional.
13. Nonprofessional may have a negative connotation and imply the individual does not do important work.
14. Nonprofessional is way too negative to be used.
15. Nonprofessional is off-putting. I've never heard the term, "paralibrarian."
16. Nonprofessional is not good.
17. Nonprofessional is not a good term as it denotes inferior value and Parlibrarian confuses the distinction between Librarians and non-librarians. Paraprofessional is too generic.
18. Nonprofessional implies negativity.
19. Nonprofessional has terrible implications.
20. Nonprofessional has a very negative connotation and does not really signify anything.
21. Nonprofessional has a negative connotation, in my opinion.
22. Nonprofessional gives a negative connotation.
23. Non-professional sounds like unprofessional—and I am very professional, and the para words sounds like I am somehow disabled.
24. Non professional needs to come off.
25. I'd find "Nonprofessional" insulting, everyone on our team is "Library Staff." "Paralibrarian" sounds strange to me.
26. I would never use the terms Paralibrarian, Paraprofessional, or nonproprofessional to describe the group of non-librarians.
27. I think the terms Paraprofessional, and Nonprofessional are demeaning!
28. I strongly dislike "nonprofessional."

Supplemental content to
J Med Libr Assoc. Jan;109(1):dx.doi.org/10.5195/jmla.2021.933
www.jmla.mlanet.org
© Schilperoort, Quezada, Lezcano 2021

29. I really don't like "Nonprofessional" I am very knowlegable but did not have the option of going to school. Para…sounds too much like paratroopers and parachutes.
30. I really dislike the term "nonprofessional". It's too similar to "unprofessional."
31. I really dislike nonprofessional—it sounds dismissive of my role.
32. I have never heard the term Paralibrarian before and I would be offended if someone called me a nonprofessional.
33. I have never heard "Nonprofessional" and think that sounds a bit harsh/bad.
34. I hate "paraprofessional", "paralibrarian", and most certainly "NONprofessional". Ugh. I want them to be not on this list.
35. I find the term "nonprofessional" suggests that I can't do the job and I dislike it.
36. I find paraprofessional and nonprofessional insulting.
37. I feel nonprofessional is completely unacceptable.
38. I don't feel as though I associate myself with non-professionals or even paraprofessionals.
39. I do not like the term Nonprofessional. It makes us sound "less than."
40. I dislike the term Nonprofessional, I'm a professional with or without a master's degree.
41. I believe some librarians treat us as if we are nonprofessional. I am not a fan of this term especially since we are usually the first people patrons approach for reference and technical help. I also get frustrated when we are asked to learn a new tool (e.g., database, citation manager) without much direction or a heads up. In other words there are a lot of decisions made without our input or any thought given to how it will impact our day to day job tasks.
42. Both Para-s are troublesome, and nonprofessional is worst of all. We had an Ivy League PhD who would've been a paraprofessional.
43. Being part library staff is a professional position, so I feel the term "nonprofessional" is inappropriate.
44. "Nonprofessional" sounds highly condescending. We are all expected to act professionally, therefore we are professionals, all of us. The "para" words annoy me less but they can definitely be used poorly in a certain context.
45. "Nonprofessional" is insulting and never acceptable.
46. 'Nonprofessional' should not be listed. That is a terrible job title.

Expressed a dislike for all or majority of terms but do not specifically state which terms.

1. They are describe what I am not. None of them describe what I am.
2. they actually all suck except for "Library Staff"—which is what everyone in the library should be called. (I hold an MLIS).
3. These are all insulting and pointless. If you want to support the staff, provide opportunities for growth and advancement within the library.
4. There is nothing for administrative positions.
5. terms don't give confidence to patrons, they often want to speak to a librarian as they expect them to be more knowledgeable even if it is a simple question.
6. Library staff.
7. I use "library staff" or "library support staff" only, not any of the other terms.
8. I find most of the terms above to be very vague and applicable to many diffferent types of library staff roles.

JMLA Journal of the Medical Library Association

Supplemental content to
J Med Libr Assoc. Jan;109(1):dx.doi.org/10.5195/jmla.2021.933
www.jmla.mlanet.org
© Schilperoort, Quezada, Lezcano 2021

Not coded to any of the 4 themes.

1. Only two are used in our library. None of the other terms that differentiate us from each other mean anything.
2. only the first two are relevant.
3. Only identify with 1 and 2.
4. None.
5. No comments.
6. No.
7. No.
8. no.
9. no.
10. No.
11. no.
12. no.
13. No.
14. No.
15. no.
16. no.
17. no.
18. no.
19. N/A.
20. I have worked in Medical Libraries for 26 years.
21. I have a Masters in Health Administration. I have worked in at a medical school for over thirty years. Why can I not move from Library staff?
22. I dislike all but the first one.
23. I am almost always called a "librarian" by our patrons and people who work outside of the library world. I always identify myself as "library staff" and not a librarian because I do not have an MLS, but pretty much everyone says "You're a librarian. You do all the same things."
24. I added Project Coordinator not just because it is my job title. I am in a big institution, and I work frequently with Project Coordinators at departments outside of the library.
